# Hemichannel composition and electrical synaptic transmission: molecular diversity and its implications for electrical rectification

**DOI:** 10.3389/fncel.2014.00324

**Published:** 2014-10-15

**Authors:** Nicolás Palacios-Prado, Wolf Huetteroth, Alberto E. Pereda

**Affiliations:** ^1^Dominick P. Purpura Department of Neuroscience, Albert Einstein College of MedicineBronx, NY, USA; ^2^Marine Biological Laboratory, Woods HoleMassachusetts, MA, USA; ^3^Department of Neurobiology, University of KonstanzKonstanz, Germany

**Keywords:** gap junction, connexin, innexin, electrical synapse, asymmetry, rectification

## Abstract

Unapposed hemichannels (HCs) formed by hexamers of gap junction proteins are now known to be involved in various cellular processes under both physiological and pathological conditions. On the other hand, less is known regarding how differences in the molecular composition of HCs impact electrical synaptic transmission between neurons when they form intercellular heterotypic gap junctions (GJs). Here we review data indicating that molecular differences between apposed HCs at electrical synapses are generally associated with rectification of electrical transmission. Furthermore, this association has been observed at both innexin and connexin (Cx) based electrical synapses. We discuss the possible molecular mechanisms underlying electrical rectification, as well as the potential contribution of intracellular soluble factors to this phenomenon. We conclude that asymmetries in molecular composition and sensitivity to cellular factors of each contributing hemichannel can profoundly influence the transmission of electrical signals, endowing electrical synapses with more complex functional properties.

## Introduction

**Figure 1:**
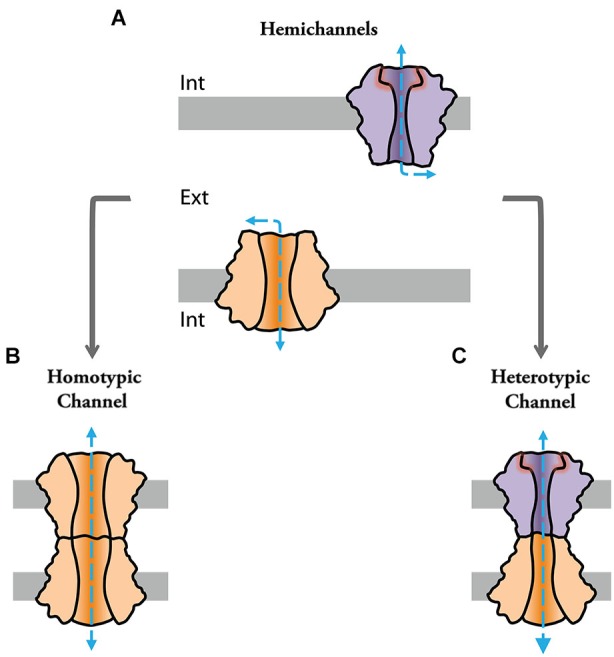
**Channels formed by connexin and innexin proteins can play functional roles as single “hemichannels” (HCs) or assemble into intercellular channels at gap junctions to provide cell-cell communication**. **(A)** Hemichannels can be molecularly different (note difference in color and shape) and act as conduits for the release of messenger molecules. **(B)** Identical HCs can assemble into intercellular “homotypic” gap junction channels. **(C)** Hemichannels of different molecular composition can assemble into intercellular “heterotypic” gap junction channels.

Channels formed by connexin (Cx) or pannexin proteins (connexon and pannexon, respectively) were shown to impact cellular properties and underlie various pathological processes by serving as conduits for ions and various autocrine and paracrine signaling molecules (Contreras et al., 2002; Bennett et al., 2003; Scemes et al., 2007; Iglesias et al., 2009; Figure 1A). Some of these channels can assemble into intercellular structures. That is, docking of two connexons or “hemichannels” (HC) from two adjacent cells form intercellular channels that cluster into structures called “gap junctions” (GJs; Goodenough and Paul, 2009; Figures 1B,C), which mediate intercellular communication between neighboring cells in virtually all tissues of deuterostomes (Hervé et al., 2005; Abascal and Zardoya, 2013). Invertebrate GJ proteins, however, are part of an unrelated gene family called innexins (Inxs; Starich et al., 1996; Ganfornina et al., 1999). Three Inx-like genes were subsequently found in the genome of vertebrates, which were named pannexins (Panxs; Panchin et al., 2000; Bruzzone et al., 2003). Interestingly, while Panxs were shown to form intercellular channels when overexpressed in oocytes (Bruzzone et al., 2003), there is little evidence so far supporting that they form GJ channels under physiological conditions (Dahl and Locovei, 2006; Sahu et al., 2014). Since Inxs form GJ channels in invertebrates (Hervé et al., 2005; Phelan, 2005), it is speculated that Panxs might have evolved to function mainly as HCs in vertebrates (Dahl and Locovei, 2006). On the other hand, recent evidence suggests that Inxs can also function as HCs or “innexons” (Dahl and Muller, 2014). Due to the current uncertainty of Panx-based GJ channels, the distinction between Inx and Panx has remained in the literature to distinguish GJ forming proteins in protostomes and cnidarians (Inx) vs. HC forming proteins in deuterostomes (Panxs) (Abascal and Zardoya, 2013).

Currently, we know that the family of Cx proteins in humans is composed by 21 genes (Söhl and Willecke, 2004) whereas Inxs represent 25 genes in *C. elegans* or eight genes in *D. melanogaster* (Adams et al., 2000; Altun et al., 2009). Gap junction channels formed by different Cxs and Inxs were shown to exhibit differential permeability and distinct electrophysiological properties providing diversity to gap junctional communication (Goodenough and Paul, 2009; Samuels et al., 2010). Notably, GJ channels can be either formed by the docking of identical (homotypic configuration; Figure 1B) or different (heterotypic configuration) HCs (Figure 1C), further enhancing the functional diversity of GJs. That is, at heterotypic channels, the molecular and functional singularities of each of the apposed/contributing HC (aHC) influence the properties of the intercellular channel and, furthermore, can potentially endow heterotypic channels with properties which could not be predicted from those displayed in homotypic configuration (Verselis et al., 1994; Oh et al., 1999). In other words, asymmetries in molecular composition of each aHC could profoundly influence intercellular communication, providing GJs with complex functional properties. In addition, many types of cells co-express several Cx or Inx isoforms with the potential to form heteromeric HCs. In the same scenario, different homomeric HCs can cluster into the same junctional plaque forming bi-homotypic GJs (Li et al., 2008), in which HCs are docked with the same kind of HC, i.e., channels are homomeric homotypic. Heterotypic/heteromeric GJ channels are likely to be present in the retina, brain and peripheral system (Söhl et al., 2000; Vaney and Weiler, 2000). Twenty-one different Cx isoforms can potentially form 210 different heterotypic GJs, however not all different Cx isoforms are compatible with each other. More than forty functional heterotypic pairs have been found and analyzed so far (Palacios-Prado and Bukauskas, 2012). Although the expression of *Drosophila* Inxs was shown to overlap in some tissues (Stebbings et al., 2000) their functional compatibility remains, in contrast to Cxs, largely unexplored.

Gap junctions constitute the basis for electrical synaptic transmission in both vertebrate and invertebrate nervous systems (Bennett and Zukin, 2004; Pereda et al., 2013). Beyond their ability to allow the passage of small messenger molecules, neuronal GJs (or “electrical synapses”) serve as low resistance pathways for the spread of electrical currents between coupled neurons, a key property for a cellular type that critically relies on electrical signaling (Bennett and Zukin, 2004; Connors and Long, 2004; Pereda et al., 2013). This article reviews data indicating that molecular differences between aHC at electrical synapses are generally associated with rectification of electrical transmission (differential resistance to current flow in one vs. the other direction between two coupled cells), a voltage-dependent property of GJ channels that has been observed at both Cx- and Inx-based electrical synapses (Furshpan and Potter, 1959; Auerbach and Bennett, 1969; Edwards et al., 1998; Rela and Szczupak, 2007; Rash et al., 2013). Here we discuss some molecular mechanisms underlying rectification of electrical transmission. We conclude that asymmetries in the molecular composition of individual HCs forming electrical synapses can strongly influence transmission of electrical signals between neurons coupled by GJs.

## Bi-directionality and symmetry of electrical transmission

**Figure 2:**
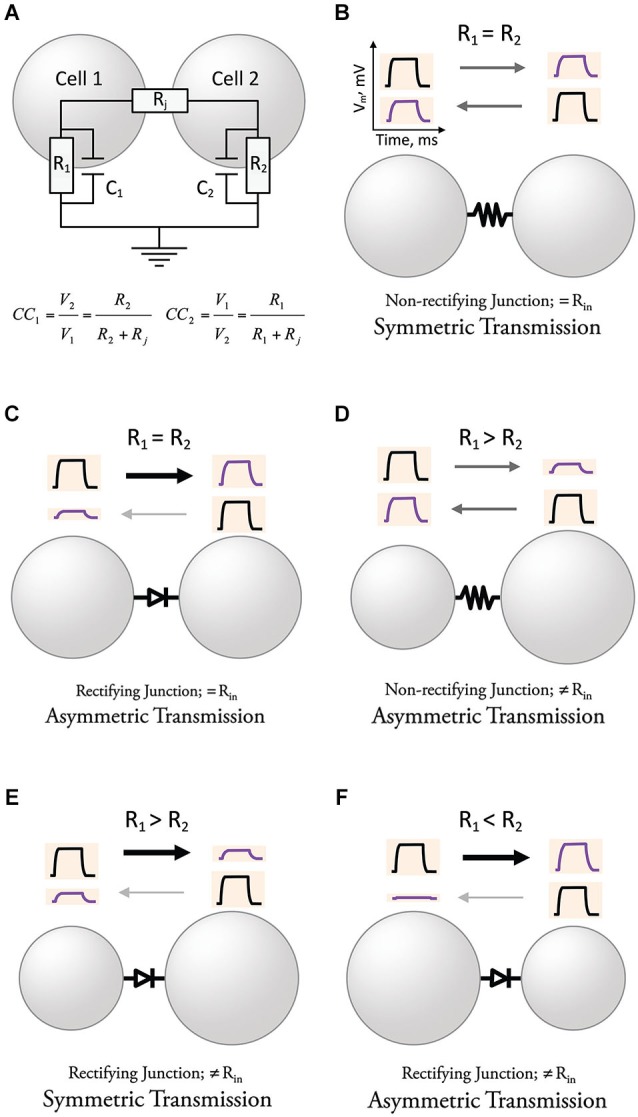
**Directionality and symmetry in electrical transmission**. **(A)** Determinants of the strength of electrical transmission. The amplitude of the coupling potential, defined by the coupling coefficients in each direction (CC_1_ & CC_2_), once the capacitance of the membrane has been charged, is determined by both the resistance of the junction (R_j_) and the resistances of the coupled cells (R_1_ & R_2_). **(B)** Electrical transmission is symmetric in cells with similar R and non-rectifying junctions (constant R_j_). **(C)** Rectifying junctions make electrical transmission between neurons with similar R asymmetric. **(D)** Cells with different R create asymmetry of electrical transmission when junctions are non-rectifying. **(E)** Electrical transmission could be symmetric in cells with different R and rectifying junctions if the effects cancel each other. **(F)** The combination of differences in R and rectifying junctions can create strong asymmetry of electrical transmission for some polarities.

Neurons operate by computing variations of the membrane potential evoked by synaptic currents and active processes, which are usually translated into trains of action potentials. The change in the membrane potential observed by the spread of presynaptic currents through GJs to a postsynaptic neuron is usually referred to as a “coupling potential”. The amplitude of this “coupling potential” does not solely depend on the conductance of GJ channels but also on the passive properties determined by the capacitance and the input resistance (R), which is directly proportional to the membrane resistance (R_m_), and indirectly proportional to the area of the membrane (size and geometry) of the coupled neurons (Figure 2A). The strength of an electrical synapse is generally expressed as the “coupling coefficient”, a ratio that expresses the amplitude of the coupling potential normalized to the amplitude of the signal that originated it in a neighboring coupled cell (see equation in Figure 2A; this value is obtained once the capacitance of the membrane is charged, which is generally referred to as “steady state”). Electrical synaptic transmission is bidirectional and symmetric when the Rs of coupled cells are similar (Figure 2B). Gap junction channels at electrical synapses were shown in some cases to behave as electrical rectifiers, that is, to offer differential resistance to the flow of currents in one vs. the other direction across the junction between two coupled neurons. As a matter of fact, the first characterization of an unequivocally electrically mediated synapse came together with the description of electrical rectification (Furshpan and Potter, 1957, 1959). Rather than a simple bidirectional spread of electrotonic potential, the crayfish giant motor synapse transmitted depolarization signals from the giant axon to the motor fiber, but not in the opposite direction. Similarly, hyperpolarization signals were transmitted only from the motor fiber to the giant axon. Although the transmission of relative positive or negative potentials is unidirectional (only in one direction), rectifying junctions allow the spread of signals in either direction. In addition, postsynaptic signals reproduced the time course of presynaptic signals, and transmission was, surprisingly, voltage-dependent, thus challenging all the criteria established for chemical transmission. The rectification properties discovered in this preparation also helped to exclude the prevailing hypothesis of gross protoplasmic connections suggested earlier to explain symmetric electrotonic spread of current between cardiac Purkinje cells (Weidmann, 1952) or among neurons of the lobster cardiac ganglion (Watanabe, 1958). Rectification was subsequently found in several electrical synapses in *in vivo* preparations exhibiting direction asymmetry of signal transfer (Smith et al., 1965; Auerbach and Bennett, 1969; Baylor and Nicholls, 1969; Ringham, 1975; Muller and Scott, 1981; Roberts et al., 1982; Margiotta and Walcott, 1983; Rash et al., 2013). Because of their properties, rectifying GJs can underlie asymmetric electrical transmission (Figure 2C). Asymmetry of electrical transmission does not necessarily require rectifying GJ channels, as differences in R of the coupled cells can make coupling coefficients stronger in the direction towards the cell with higher R (Figure 2D, Trenholm et al., 2013). Moreover, rectifying junctions can make a synapse bi-directional by counterbalancing the effect of differences in R of the coupled neurons (Figure 2E). Finally, the combination of rectifying junctions and differences in R of coupled cells can create strong asymmetric transmission (Figure 2F). Thus, although intimately related, directionality, rectification, and symmetry express different properties of electrical synaptic transmission and should not be considered interchangeable. In other words, electrical transmission could be: (1) bi-directional and asymmetric; (2) non-rectifying and markedly asymmetric; and (3) bidirectional and rectifying. Finally, while directionality and symmetry refer to electrical transmission (coupling potentials), the term rectification should be reserved to describe the asymmetric transjunctional current-voltage relationship of certain GJ channels.

## Asymmetry in hemichannel composition is associated with rectification of electrical transmission

The association of asymmetry of hemichannel composition and electrical rectification has been observed at both Inx- and Cx-based electrical synapses.

### Innexin-based electrical synapses

It was actually proposed that the formation of homomeric, homotypic channels is generally rare among fly Inxs (Phelan and Starich, 2001). Of the eight Inxs in *Drosophila*, a few were shown to form heterotypic GJs (reviewed in Hasegawa and Turnbull, 2014). Most information about electrical properties of heterotypic GJs in the fly brain exists for splice variants of the gene *shakingB*. Here we focus on two different circuits that both include shakB-based heterotypic electrical synapses in their architecture, the giant fiber system (GFS) and the antennal lobe. One forms a rather rigid reflex network, the other is a heavily modulated sensory information processor dealing with odors. In addition, we discuss the contribution of heterotypic GJs to memory formation in the fly brain and to *C. elegans* nervous system signaling.

The most complete picture of GJ function within a behavioral circuit is based on the GFS in *Drosophila*, an efficient escape reflex circuit that initiates a “jump and flight” response (reviewed in Allen et al., 2006). Threatening sudden stimuli can evoke a response in the giant fiber (GF) via giant commissural interneurons in the brain. The GF provides a fast connection of the brain with the ventral nerve cord in the thorax, where it forms mixed electrical and chemical synapses onto two cell types, a motor neuron that innervates a contralateral leg muscle, and an interneuron, which activates ipsilateral flight muscle motor neurons (Blagburn et al., 1999). The first evidence of the presence of GJs in this network stems from intracellular recordings on the GF with simultaneous brain stimulation and flight muscle recordings (Tanouye and Wyman, 1980). Subsequent work on the mutants *shaking-B*^2^ (*shakB*^2^) and *passover* characterized the involved gene (Thomas and Wyman, 1984; Phelan et al., 1996; Sun and Wyman, 1996). It was later discovered that this *shakB* (or *inx8*) gene gives rise to five different splice variants, resulting in three confirmed protein isoforms: ShakB(Lethal), ShakB(Neural), and ShakB(Neural+16), which has 16 more amino acids on the amino-terminus (Zhang et al., 1999). Interestingly, while ShakB(Lethal) is capable of functional homotypic channel formation in *Xenopus* oocytes, the ShakB(Neural) variant is not (Phelan et al., 1998). Ten years later the same lab used the GFS to provide first evidence that electrical rectification emerges from differential aHC composition and formation of heterotypic GJs (Phelan et al., 2008). They identified two ShakB variants being responsible for heterotypic GJs in the GFS: ShakB(Neural+16) in the presynaptic GF, and ShakB(Lethal) in the postsynaptic motor neuron and interneuron. A more recent study using oocytic expression of chimeric ShakB proteins suggested a role for the amino-terminal end of ShakB in voltage gating and electrical rectification (Marks and Skerrett, 2014).

Heterotypic GJs involving ShakB are also found in the antennal lobe. The antennal lobe is the first integration center of olfactory information in insects and shows structural similarity to the vertebrate olfactory bulb; within neuropilar substructures called glomeruli, the olfactory sensory neurons converge onto projection neurons (PNs), which in turn relay the olfactory information into higher brain regions (Wilson, 2013). The glomeruli are interconnected by a third class of antennal lobe neurons, the amacrine local interneurons (LNs). The majority are GABAergic and mainly provide lateral inhibition on the presynaptic terminal of the sensory neurons (iLNs), but some are excitatory (eLNs) and either cholinergic (Shang et al., 2007) or glutamatergic (Chou et al., 2010; Das et al., 2011). The cholinergic eLNs form chemical synapses onto iLNs and electrical synapses with PNs (Huang et al., 2010; Yaksi and Wilson, 2010). The mutant *shakB^2^* abolishes electrical transmission between eLNs and PNs, and RT-PCR identified shakB transcripts in PNs (Yaksi and Wilson, 2010). *ShakB*^2^ (which affects both Neural variants) was successfully rescued by ectopic expression of ShakB(Neural) in adult flies. Since homomeric ShakB(Neural) HCs fail to form functional GJs (Phelan et al., 1998; Curtin et al., 2002), heterotypic interaction with another Inx, probably ShakB(Lethal) like in the GFS circuit, seems likely. A more targeted rescue in either PNs or eLNs will resolve on which side the ShakB(Neural) HC is essential; based on the electrical properties of the junction it would be expected on the eLN side.

There is also evidence for the presence of heterotypic GJs in the mushroom body (MB). The MB is regarded as the homologous structure of the vertebrate pallium in the brain of protostomes (Tomer et al., 2010) and is crucial for associative memory processes (Perisse et al., 2013). This paired neuropil consists mainly of about 2000 Kenyon cells on each side, which can roughly be subdivided in a dendritic calyx region, two orthogonal, elongated lobes, which contain the majority of presynaptic sites, and a peduncle that connects calyx with lobes. Prevailing sensory input to the *Drosophila* calyx is of olfactory nature, coming from the antennal lobe. Certain Kenyon cell subdivisions exhibit preferential roles in memory acquisition and in memory retrieval. The MB is innervated by two large amacrine cells per hemisphere; the GABAergic anterior paired lateral cell (APL), which innervates all MB regions (Liu and Davis, 2009), and the serotonergic dorsal paired medial cell (DPM) which innervates peduncle and lobes only (Lee et al., 2011). A prominent role of the APL is to maintain signal sparseness by feedback inhibition (Lin et al., 2014), but it also shows involvement in labile appetitive memory (Pitman et al., 2011). The DPM is crucial for long-term memory consolidation, so its role can be separated from APL (Pitman et al., 2011). Both neurons seem to be electrically connected by heterotypic channels formed by Inx6 (DPM) and Inx7 (APL), especially in a subregion of the MB. This was inferred from contact marker expression and a combination of immunostainings, targeted RNAi expression against various Inxs in DPM and APL, dye coupling backfills and behavioral experiments (Wu et al., 2011; Pitman et al., 2011). Taken together, it is tempting to speculate that a rectifying electrical synapse between APL and DPM could contribute to generate a reverberant circuit, thus providing the ongoing cellular activity to consolidate a memory trace. The presence of this putative heterotypic channel is interesting for several reasons: it involves a novel pair of interacting Inxs, despite bigger spatial overlap between both contributing cells it seems to be segregated to a specific subcellular region, and, importantly, because of its potential contribution to a memory consolidation process.

The existence of numerous heterotypic GJs was suggested also to be the case for *C. elegans* Inxs, with the notable exception of UNC-7 and UNC-9, and possibly Inx14 with Inx8 or Inx9 (Simonsen et al., 2014). The *unc-7* gene gives rise to three protein isoforms, and the homomeric heterotypic channel formed by the UNC-7S (or UNC-7b) isoform and UNC-9 was shown to be rectifying (Starich et al., 2009). Since both Inxs are widely expressed in nerve cells (Altun et al., 2009), and ~10% of all synapses in *C. elegans* are electrical, this rectifying synapse might contribute significantly to direct signal transduction in the nematode nervous system. This is supported by the locomotion phenotype in mutants of both *unc-7* and *unc-9* (Starich et al., 1993; Barnes and Hekimi, 1997).

Gregarious behavior in *C. elegans* is determined by sensory integration in a hub-and-spoke circuit where the RMG neuron forms electrical synapses with many sensory neurons (Macosko et al., 2009). Activation or inhibition of this sensory integration induces gathering or solitary behaviors, respectively. Interestingly, RMG neurons are only labeled with the *unc-7a* promoter fragment while its sensory partners express different Inxs: IL2, ADL and AWB express UNC-9; IL2, ADL and ASK express Inx-18; and IL2, ADL and ASH express Inx-19 (Altun et al., 2009). Since UNC-7S and UNC-9 are known to form rectifying heterotypic junctions (Starich et al., 2009), it is possible that electrical rectification is involved in sensory integration in the RMG hub-and-spoke circuit, and therefore heterotypic GJ might be involved in gregarious behavior of *C. elegans*. This type of circuit motif—one integrating hub neuron connected to many sensory neurons by electrical synapses—are present in large numbers (more than 15 different hubs) in the nematode nervous system and may be a conserved functional unit for coincidence detection (Rabinowitch et al., 2013). Finally, although the relationship with heterotypic Inx-based GJs still needs to be established, multiple rectifying electrical synapses have been described in various invertebrates, such as crayfish (Furshpan and Potter, 1959), horseshoe crab (Smith et al., 1965) and leech (Baylor and Nicholls, 1969; Muller and Scott, 1981).

### Connexin-based electrical synapses

Although the presence of asymmetric transmission has been reported at electrical synapses between several vertebrate cell types, such as the inferior olive (Devor and Yarom, 2002), striatum (Venance et al., 2004), cochlear nucleus (Apostolides and Trussell, 2013) and thalamus (Haas et al., 2011) and reportedly involving in some of these cases asymmetry of GJ conductance (Devor and Yarom, 2002; Venance et al., 2004; Haas et al., 2011), electrical rectification was demonstrated in only a few cases (Auerbach and Bennett, 1969; Ringham, 1975; Rash et al., 2013). Recent evidence suggests that, as observed in invertebrates, electrical rectification is also associated with asymmetry in the molecular composition of aHCs. That is, electrical synapses at auditory afferents and the teleost Mauthner cell known as “Club endings” are formed by two homologs of mammalian Cx36 (considered the main synaptic Cx in mammals due to its widespread expression in neurons (Condorelli et al., 2000)), Cx35 and Cx34.7 (Rash et al., 2013). As a result of additional genome duplication (Volff, 2005), teleost fish have more than one homologous gene for most mammalian Cxs (Eastman et al., 2006). Remarkably, while Cx35 is restricted to presynaptic GJ hemiplaques (the portion of the GJ plaque contributed by each cell), Cx34.7 is restricted to postsynaptic hemiplaques, forming heterotypic junctions (Rash et al., 2013). In contrast to many different Cxs that are compatible to form heterotypic GJs, Cx36 is known so far to form only “homotypic” GJs (Teubner et al., 2000; Li et al., 2004). From an evolutionary point of view, the existence of compatible Cx36 teleost homologs that form heterotypic channels provided neurons with the ability to connect through GJs with more complex properties. Estimates of junctional conductance (*g*_j_) between Club endings and the Mauthner cell revealed a four-fold difference between the antidromic (from the postsynaptic Mauthner cell to the presynaptic Club ending) and orthodromic (from the Club ending to the Mauthner cell) directions (Rash et al., 2013). This rectifying property is thought to play an important functional role by promoting cooperativity between different auditory afferents (see below).

## Mechanisms underlying rectification of electrical transmission

### Gap junctions as diodes

The original mechanism proposed for rectification of electrical transmission was represented as a simple analogy to an electric rectifier or diode (Furshpan and Potter, 1959), in which separation of negative and positive permanent charges results in an asymmetric energy barrier. This barrier generates instantaneous transjunctional current (*I*_j_) rectification with characteristics of a p-n junction in semiconductors. At that time GJ channels had not yet been discovered and thus the properties of the rectifier and the electrostatic effect were assigned to the “synaptic membrane”. Nonetheless, the novel hypothesis of p-n junctions in biological membranes was examined (Mauro, 1962; Coster, 1965), and provided a theoretical framework for considering fixed charges in junctional membranes (Brink and Dewey, 1980) that could explain the steep rectification of the junctional conductance-voltage relation (*g*_j_-*V*_j_) in some electrical synapses.

The hypothesis that electrical rectification could arise from an asymmetry in aHC composition came in the late 70’s (Bennett, 1977; Loewenstein, 1981). With the exogenous expression of different Cx isoforms, it was possible to examine this hypothesis. Indeed, electrical rectification of heterotypic GJ channels (originally called heteromolecular or hybrid cell-cell channels) was first studied in pair of oocytes overexpressing Cx32/Cx26 or Cx32/Cx43 GJs (Swenson et al., 1989; Werner et al., 1989; Barrio et al., 1991). In the case of heterotypic Cx32/Cx26 GJ channels, asymmetries in the instantaneous and steady-state *g*_j_-*V*_j_ relationships were observed (Barrio et al., 1991). To make a clear distinction between instantaneous and steady-state asymmetries in the* g*_j_-*V*_j_ relationship, we refer to instantaneous and steady-state asymmetries as “electrical rectification” and “asymmetric gating”, respectively.

**Figure 3:**
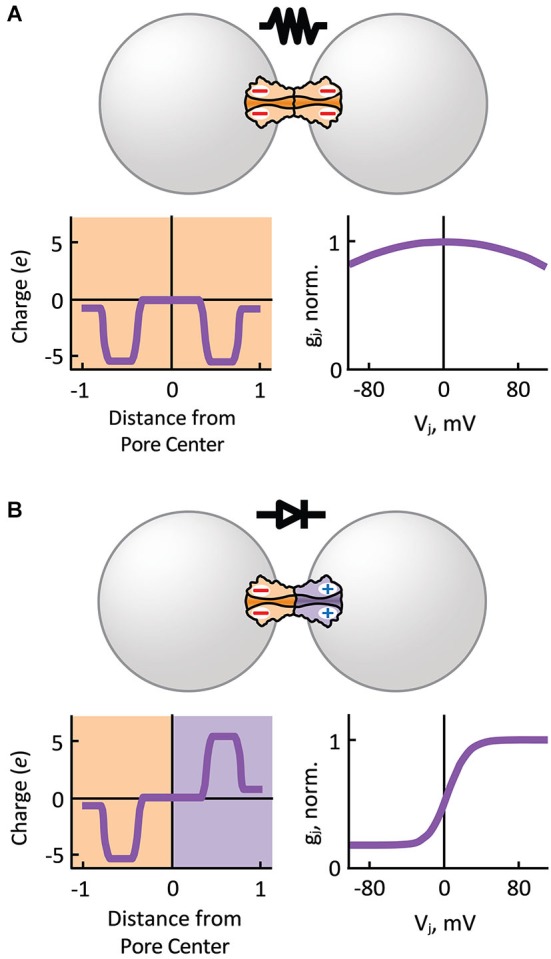
**Hemichannel composition determines the symmetry of electrical transmission**.** (A)** Homotypic gap junction channels that comprise symmetric charge distribution with respect to the pore center of the channel, behave as passive resistors with symmetric junctional conductance (*g*_j_) over transjunctional voltage (*V*_j_) dependence (normalized to *g*_j_ value at *V*_j_ equal zero). **(B)** Heterotypic gap junction channels that comprise an asymmetry in positive and negative charge distribution with respect to the pore center of the channel behave as electrical rectifiers (p-n junction) with steep asymmetric *g*_j_-*V*_j_ dependence. Normally, depolarizing (positive) potentials are more easily transmitted from the cell with negatively charged HCs to the cell with positively charged HCs.

Based on single GJ channel and HC recordings showing multiple *I*_j_ substates, we know that the steady-state *I*_j_-*V*_j_ relationship of GJ channels is the product of two *V*_j_-sensitive gating mechanisms present in each aHC, the *fast* or “*V*_j_” gate and the *slow* or “loop” gate (Bukauskas and Verselis, 2004). The probability of each *V*_j_-sensitive gate to dwell in a closed state is a function of the intensity and relative polarity of *V*_j_ (gating polarity). The instantaneous *I*_j_-*V*_j_ relationship is mostly determined by the electrical properties of the unitary conductance of the fully open state (γ_o_) of GJ channels, which can rectify by allowing larger *I*_j_s in one direction than in the other. The unitary conductance of the residual state (γ_res_; one or two *fast* gates in closed position) may also rectify (Bukauskas et al., 1995; Oh et al., 1999), and therefore can contribute to electrical rectification. In general, homotypic GJs show symmetric *g*_j_-*V*_j_ relationships for either polarity of *V*_j_ (Figure 3A). However, asymmetry in the composition of aHCs (or transjunctional asymmetry in cytosolic factors; see below) may result in electrical rectification (Figure 3B) and asymmetric gating.

The mechanism for asymmetric gating observed in Cx32/Cx26 GJ channels was explained by a difference in gating polarity of voltage-sensitive gates present in Cx26 and Cx32 aHCs (Verselis et al., 1994). Heterotypic GJs that possess aHC with opposite gating polarity exhibit marked asymmetric gating since one polarity of *V*_j_ simultaneously opens the gates in both aHCs, and the opposite polarity closes them. In addition to opposite gating polarity, asymmetric gating can also be produced by differences in unitary conductances of aHCs (γ_o,H_), or simply by differences in intrinsic sensitivity to *V*_j_ (Bukauskas et al., 1995; Rackauskas et al., 2007). When γ_o,H_s are considerably dissimilar, like in the case of Cx43/Cx45 heterotypic GJs (γ_o,H_ of Cx43 is ~4 times higher than that of Cx45), a bigger fraction of *V*_j_ drops across the aHC with higher resistance (Cx45), thus enhancing its sensitivity to *V*_j_ compared to the aHC with smaller resistance (Cx43). Therefore, differential drop of *V*_j_ in aHCs of heterotypic GJs may also result in asymmetric gating (Bukauskas et al., 2002). Although asymmetric gating may be important to determine *I*_j_ and transjunctional flux directionalities under long-lasting asymmetries in *V*_j_ (Palacios-Prado and Bukauskas, 2009), electrical rectification (instantaneous asymmetry) determines the directionality of synaptic electrical transmission between neurons with brief (millisecond) oscillatory changes in *V*_j_ during action potentials.

**Figure 4:**
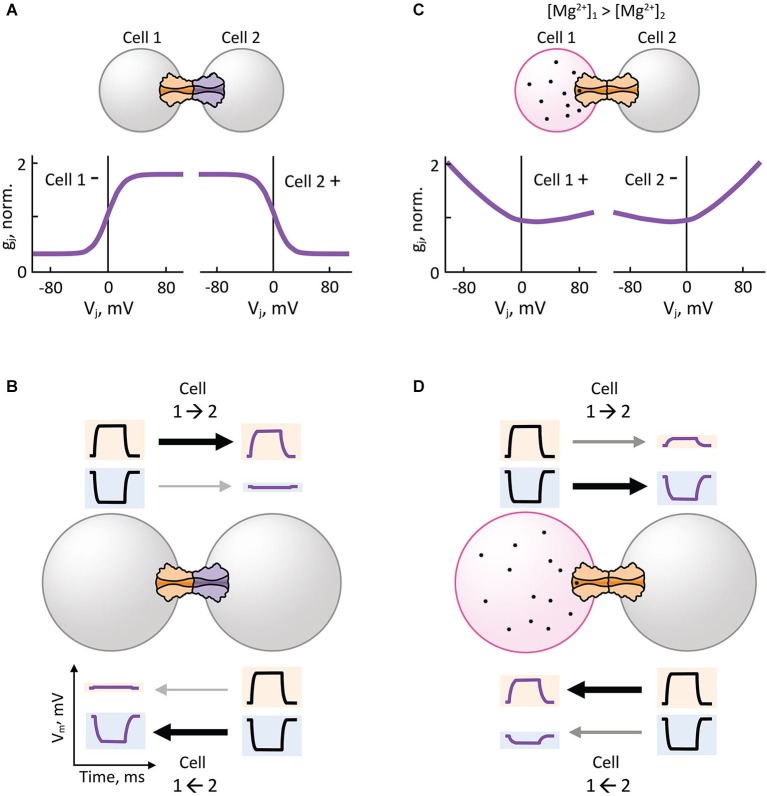
**Hemichannel composition and intercellular gradient of charged cytosolic factors can lead to rectification of electrical transmission**. **(A,B)** Heterotypic gap junction channels with steep asymmetric *g*_j_-*V*_j_ dependence **(A)** facilitate or attenuate the electrical transmission of depolarizing (positive) potentials from Cell 1 to Cell 2 (1→2) or 1←2, respectively **(B)**. The same junctions facilitate or attenuate the electrical transmission of hyperpolarizing (negative) potentials from 1←2 or 1→2, respectively **(B)**. **(C,D)** Transjunctional gradient of free magnesium ion concentration ([Mg^2+^]_i_) induces asymmetric* g*_j_-*V*_j_ dependence in homotypic gap junction channels **(C)** that are hypersensitive to [Mg^2+^]_i_, such as Cx36 gap junction channels. Electrical transmission of depolarizing potentials is facilitated from 2→1 **(D)**, which is the opposite direction of the transjunctional [Mg^2+^]_i_ gradient (1→2). The same transjunctional [Mg^2+^]_i_ gradient facilitates the electrical transmission of hyperpolarizing potentials from 1→2 **(D)**.

Analysis of heterotypic Cx32/Cx26 GJs at the single channel level revealed that γ_o_ rectifies depending on *V*_j_ (Bukauskas et al., 1995). Using this premise, an electrodiffusive model that solves the Poisson-Nernst-Planck (PNP) equations in one dimension (Chen and Eisenberg, 1993) was used to describe the asymmetric single channel fluxes and currents observed in heterotypic Cx32/Cx26 GJs (Oh et al., 1999). The PNP model in combination with site-directed mutagenesis successfully predicted that electrical rectification was produced by an asymmetric position of fixed charged amino acid residues present in the heterotypic Cx32/Cx26 channel pore. These findings demonstrated that the original diode hypothesis of p-n junctions could indeed generate electrical rectification of synaptic transmission based on the asymmetric position of charges near the channel-pore surface (Figure 3B) that, in turn, produce differences in ionic conductance and selectivity of HCs (Suchyna et al., 1999). Thus, heterotypic GJ channels that form rectifying junctions with steep asymmetric *g*_j_-*V*_j_ relationship (Figure 4A) can make an electrical synapse nearly unidirectional by allowing the transmission of depolarizing and hyperpolarizing potentials in only one direction (opposite to each other), and restricting the transmission of depolarizing and hyperpolarizing potentials in the opposite direction (Figure 4B). The mechanism for electrical rectification and asymmetric gating observed in the *Drosophila* GFS is indeed associated with molecular asymmetry of HCs (Phelan et al., 2008). Since ShakB(Neural+16) and ShakB(Lethal) variants exhibit significant differences in amino acid sequence and sensitivity to *V*_j_, it is likely that electrical rectification arises from asymmetry in position of charges (p-n junction), and asymmetric gating arises from differences in intrinsic *V*_j_-sensitivity of aHCs rather than opposite gating polarities or differences in γ_o,H_.

In addition to the p-n junction hypothesis for electrical rectification, a unique voltage-dependent gating mechanism was proposed after a detailed characterization of the rectifying crayfish giant motor synapse using high-quality voltage clamp at low temperatures (Jaslove and Brink, 1986). These studies suggested that, rather than an instantaneous electrostatic effect, the rectification profile of the *I*_j_-*V*_j_ relationship contained a voltage-dependent kinetic component with a time constant in the order of milliseconds, which was attributed to a rapid gating mechanism present in one of the aHCs. The authors proposed that this gate was set to a low open probability at resting conditions and that changing the polarity of *V*_j_ would rapidly open the gates. This “millisecond timescale” gating mechanism has not been reported in any other Cx- or Inx-based rectifying electrical synapse; hence it is unclear whether rapid gating mechanisms may contribute to the observed electrical rectification in other invertebrate and vertebrate electrical synapses.

Gap junctions can occur in homocellular or heterocellular junctions; that is, coupled cells can be from the same or different cell types and perform similar or different functions, respectively. One remarkable similarity among electrical synapses showing steep rectification is that they occur mostly in heterocellular junctions and very often there is a difference in the resting potential of coupled neurons that give rise to a relatively constant *V*_j_ (Giaume and Korn, 1983; Ramón and Rivera, 1986). Regardless of the mechanism of rectification, the resting *V*_j_ derived from the difference in the resting potential of neurons forming rectifying electrical synapses in crayfish and leech is essential to produce steep rectification, since bidirectional transmission of depolarization pulses could be achieved by reversing the resting *V*_j_ polarity (Giaume et al., 1987; Rela and Szczupak, 2007). Both p-n junction and rapid gating mechanisms imply a molecular asymmetry in aHC composition, and both require a resting *V*_j_ (difference in membrane potentials between the coupled cells) to exhibit significant electrical rectification or asymmetric gating, respectively. As an analogy to p-n junctions in semiconductors and silica nanochannels (Cheng and Guo, 2007), the resting *V*_j_ would normally set GJs to a low conductive state by producing a “reversed bias” effect (expression used when the flow of current is obstructed by increasing the resistance). Only action potentials that lower this resting *V*_j_ would produce a “forward bias” effect in the junction to allow the spread of electrotonic potentials.

### Contribution of intracellular soluble factors

Gap junction channels and HCs are highly regulated according to cellular requirements and respond to various changes in the extracellular and intracellular environments. Besides their sensitivity to *V*_j_, GJ channels and HCs are sensitive to phosphorylation, lipophilic molecules and other chemical factors (Baldridge et al., 1987; Bennett et al., 1991; Harris, 2001; Bukauskas and Verselis, 2004; Jouhou et al., 2007; Márquez-Rosado et al., 2012). Furthermore, GJ channels are sensitive to changes in intracellular ionic composition, such as intracellular pH, Mg^2+^ and Ca^2+^ that may vary under physiological conditions (Noma and Tsuboi, 1987; Cheng and Reynolds, 2000; Chesler, 2003; Matsuda et al., 2010; Shindo et al., 2010; Yamanaka et al., 2013). This suggests that modulation of electrical and metabolic gap junctional intercellular communication by these factors may be important for normal cell function.

It has been recently reported that Cx36-containing electrical synapses expressed in the mesencephalic nucleus of the trigeminal nerve (MesV) and the thalamic reticular nucleus (TRN) as well as heterologous expression systems transfected with Cx36 are bi-directionally modulated by changes in intracellular concentration of free Mg^2+^ ([Mg^2+^]_i_) (Palacios-Prado et al., 2013, 2014). This is a novel Mg^2+^-dependent form of electrical synaptic plasticity where *g*_j_ can be augmented or reduced by lowering or increasing [Mg^2+^]_i_, respectively. These studies support the notion that [Mg^2+^]_i_ controls neuronal coupling via modulation of gating mechanisms of Cx36 GJs by interacting with a Mg^2+^-sensitive domain located in the lumen of the GJ channel. Since intracellular levels of ATP determines [Mg^2+^]_i_ (Lüthi et al., 1999), Mg^2+^-dependent plasticity of electrical synapses could be under control of neuronal metabolism and circadian rhythms (Dworak et al., 2010). In addition, electrical synaptic transmission could potentially decrease after neuronal depolarization and glutamate exposure, due to an increment in [Mg^2+^]_i_ (Kato et al., 1998; Shindo et al., 2010).

Electrical synapses formed by Cx36 show a unique Mg^2+^-dependent instantaneous *g*_j_-*V*_j_ relationship, in which instantaneous *g*_j_ increases over *V*_j_ under high [Mg^2+^]_i_, or remain constant over *V*_j_ under low [Mg^2+^]_i_. Interestingly, an intercellular gradient of Mg^2+^ (asymmetric transjunctional [Mg^2+^]_i_) produces electrical rectification (Figure 4C) and asymmetric gating in homotypic GJs by affecting the instantaneous and steady-state *g_j_-*V*_j_* relationship of Cx36, respectively (Palacios-Prado et al., 2013, 2014). Asymmetric transjunctional [Mg^2+^]_i_ produces greater transmission of depolarizing or hyperpolarizing potentials from the cell with lower or higher [Mg^2+^]_i_, respectively, compared to the opposite directions (Figure 4D). To explain this unique electrical rectification of Cx36, the authors proposed that a combination of two or more mechanisms are necessary: asymmetric fixed charges inside the Cx36 aHC pore that produce a p-n junction type of rectification; and a *V*_j_-dependent modulation of Mg^2+^ interaction with its binding sites inside the pore. Mg^2+^-dependent plasticity of Cx36 GJ channel properties is the only described mechanism so far by which transjunctional asymmetry is derived from a diffusible cytosolic factor that produces electrical rectification in homotypic GJs; all other examples arise from a molecular asymmetry in aHC composition. In principle, transjunctional asymmetry in ATP concentration may also induce electrical rectification by producing a transjunctional asymmetry in [Mg^2+^]_i_. It is noteworthy that other intracellular diffusible cations such as H^+^, Ca^2+^ and spermine have been shown to affect cell-cell coupling via gating mechanisms in a Cx-specific manner (White et al., 1990; Musa et al., 2004; Harris and Contreras, 2014), but their effect on electrical rectification is yet to be demonstrated.

Asymmetry in the molecular composition of aHCs can also play a role in the effects of cytosolic factors. Heterotypic channels formed by expression of Cx35 and Cx34.7 in cell lines (the Cxs present at Club ending-Mauthner cell synapses) exhibited differential sensitivity to changes in [Mg^2+^]_i_, suggesting that molecular differences in heterotypic junctions might also contribute to generate electrical rectification by expressing a differential sensitivity to cytosolic factors (Rash et al., 2013).

## Functional properties of rectifying electrical synapses

Rectifying electrical synapses have been proposed to play important functional roles within various neuronal networks (Furshpan and Potter, 1957; Edwards et al., 1999; Allen et al., 2006; Gutierrez and Marder, 2013). Providing directionality to electrical transmission between pre- and postsynaptic neurons, rectifying electrical synapses can significantly contribute to general signal transduction as in *C. elegans* (Starich et al., 2009) and are a feature in many escape networks (Furshpan and Potter, 1959; Edwards et al., 1999; Allen et al., 2006; Phelan et al., 2008). Rectifying electrical synapses were initially described at the giant motor synapses of the abdominal nerve cord of the crayfish between GFs and giant motor axons that innervate the flexor musculature of the tail (Furshpan and Potter, 1959). They also mediate directional communication in the *Drosophila* GFS (Allen et al., 2006) and between mechanoreceptor afferents and interneurons synapsing on the lateral giant neurons in crayfish (Edwards et al., 1999). Their ability to generate voltage-dependent directional transmission was also reported to be advantageous for certain motor behaviors in leech (Rela and Szczupak, 2003) and in fish spinal cord (Auerbach and Bennett, 1969), providing fast directional communication between identifiable interneurons and motor neurons.

Interestingly, rectifying electrical synapses can also underlie bidirectional communication between neuronal processes of dissimilar size, compensating for unfavorable electrical and geometrical conditions for the symmetrical spread of currents through the junctions. This is the case of a group of identifiable auditory synapses on the Mauthner cell known as Club endings (Pereda et al., 2004); the Mauthner cell network mediates auditory-evoked escape responses in fish (Faber and Pereda, 2011). Because electrical synapses at Club endings are bidirectional, the signals produced by a population of active Club endings in the Mauthner cell dendrite can influence the excitability of non-active neighboring Club endings, thus serving as a mechanism for “lateral excitation” (Pereda et al., 1995). Lateral excitation increases the sensitivity of sensory inputs (Herberholz et al., 2002). Electrical rectification favors this mechanism of lateral excitation by promoting the spread of currents originated in the dendrite to the presynaptic afferents, which otherwise would passively spread towards the lower input resistance soma of the Mauthner cell (Rash et al., 2013). Thus, by favoring the spread of currents to the presynaptic afferent, the rectification properties of electrical synapses between Club endings and the Mauthner cell enhance bi-directionality of electrical communication between these two cells of dissimilar size and geometry. From the functional point of view, lateral excitation promotes the coordinated activity of a population of Club endings, thus increasing the efficacy of the auditory input for the initiation of an escape response.

Recent studies suggest that rectifying electrical synapses are capable on endowing networks with more complex behaviors. Modeling studies explored the impact of rectifying electrical synapses in a pattern-generating neuronal network containing both chemical and electrical synapses (Gutierrez and Marder, 2013). The presence of rectifying electrical synapses was observed to have profound functional consequences, altering the sensitivity of the network dynamics to variations in the strength of chemical synapses (Gutierrez and Marder, 2013). Remarkably, the addition of rectifying electrical synapses to certain network configurations yielded robust circuit dynamics that were insensitive to variations in the strength of chemical synapses (Gutierrez and Marder, 2013), suggesting that the presence of rectifying electrical synapses is likely to play important roles in the stability and function of neural networks.

Finally, as a result on their voltage-dependent properties, rectifying electrical synapses were proposed to act as coincidence detectors (Edwards et al., 1998; Marder, 2009). Coincidence detection is an essential property of all nervous systems and is sustained by a variety of molecular, cellular and network properties. This phenomenon has been implicated in visual perception (Veruki and Hartveit, 2002), sound source localization (Joris et al., 1998), memory formation (Tsien, 2000), and motor control (Hjorth et al., 2009), amongst others. While non-rectifying electrical synapses are considered coincidence detectors of inputs arriving simultaneously at two different coupled neurons (Galarreta and Hestrin, 2001; Veruki and Hartveit, 2002), electrical rectification underlies the ability of the lateral giant neurons of crayfish to sum inputs that arrive synchronously (Edwards et al., 1998). Remarkably, this mechanism provides a significant temporal fidelity and it does not operate for inputs that are separated by only 100 ms or more. Because rectifying synapses in this system only allow bidirectional current flow when the presynaptic afferents are depolarized relative to the postsynaptic compartment (the lateral giant neuron), current flows increase during the presynaptic spike and remain electrically coupled after its completion (Edwards et al., 1998). Taking advantage of this property, synchronous inputs from mechanoreceptor afferents and interneurons integrate effectively and produce large excitatory responses. Asynchronous inputs, on the other hand, are much less efficient in activating the mechanism because: (1) the early arriving postsynaptic potential retards the opening of voltage-sensitive channels at additional synapses; and (2) the late arriving synaptic currents are shunted by the increase in *g*_j_. Given the involvement of these neurons in escape responses, the coincidence detection mediated by the voltage-dependent properties of rectifying electrical synapses allows crayfish to elicit reflex escape responses only to particularly abrupt mechanical stimuli (Edwards et al., 1998).

## Conclusions

**Figure 5:**
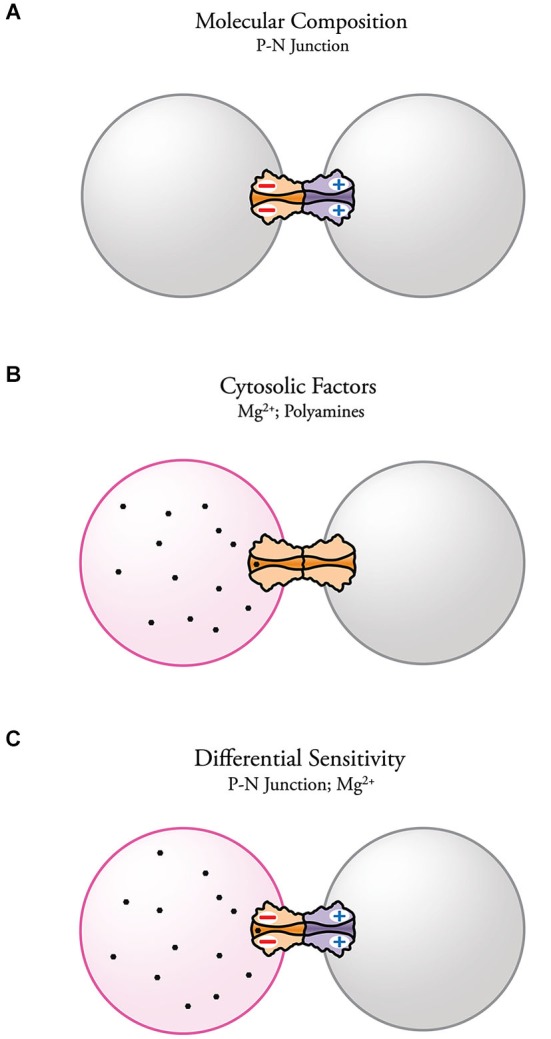
**Possible mechanisms underlying electrical rectification at electrical synapses**. **(A)** Steep electrical rectification can be attainable by the separation of fixed positive and negative charges at opposite ends of heterotypic gap junction channels (p-n junction) resulting from asymmetries in the molecular composition of the HCs. **(B)** Electrical rectification can also result from the presence of an intercellular gradient of charged cytosolic factors, such as Mg^2+^ and spermine, which alter channel conductance. Molecular diversity can make some channels more susceptible of interacting with certain cytosolic factors. **(C)** Complex electrical rectification can arise from the combination of both mechanisms.

Electrical transmission has become a topic of high interest in neuroscience. Together with the already established role of electrical synapses in invertebrates and cold-blooded vertebrates, evidence for the presence and importance of electrical synapses in the diverse areas of the mammalian brain continues to increase. Despite their wide distribution and functional relevance, the molecular complexity of electrical synapses and how this complexity affects synaptic function is still poorly understood. The evidence reviewed here indicates that the molecular composition of each aHC can endow neuronal GJs with important functional properties. More specifically, asymmetry in the molecular composition of aHCs has been associated with rectification of electrical transmission. The fact that such association was found at both Inx- and Cx-based electrical synapses emphasizes the contribution of the molecular asymmetry in underlying this voltage-dependent phenomenon. It has been shown that heterotypic channels with asymmetric position of charges near the channel-pore surface act as p-n junctions (diode hypothesis) with asymmetric transjunctional current flow (Figure 5A). Electrical rectification can also be observed at homotypic channels, arising from transjunctional asymmetries in the concentration of cytosolic factors that are capable of interacting with the channel pore (Figure 5B). Finally, cytosolic factors can contribute to electrical rectification at heterotypic junctions if one of the aHCs is more susceptible to interact with them, further enhancing the rectifying properties of the junction (Figure 5C).

Despite the presence of molecularly distinct pre- and postsynaptic sites, chemical synapses are considered indivisible functional units at which both sites are required to generate synaptic function. Electrical synapses can be viewed in a similar way. The fact that the docking of two HC is required does not necessarily imply that their molecular composition and that of the hemiplaques are the same. Hemiplaques should be different, suggesting that electrical synapses in analogy to chemical synapses can have distinct pre- and postsynaptic sites, endowing electrical synapses with more complex functional properties. While we emphasize in this article asymmetries in the composition of aHCs by GJ-forming proteins, asymmetries might also include the presence of associated scaffolding and regulatory proteins. Finally, an interesting scenario would be if asymmetries could be dynamically created by post-translational modifications of Cxs in only one of the aHCs (asymmetric phosphorylation), or by differences in the intracellular concentration of soluble factors that affect channels properties as a result of metabolic changes in one of the coupled cells, providing electrical synapses with plastic rectifying properties.

## Conflict of interest statement

The authors declare that the research was conducted in the absence of any commercial or financial relationships that could be construed as a potential conflict of interest.
